# Exercise, disease state and sex influence the beneficial effects of Fn14-depletion on survival and muscle pathology in the *SOD1*^*G93A*^ amyotrophic lateral sclerosis (ALS) mouse model

**DOI:** 10.1186/s13395-024-00356-0

**Published:** 2024-10-14

**Authors:** Gareth Hazell, Eve McCallion, Nina Ahlskog, Emma R. Sutton, Magnus Okoh, Emad I. H. Shaqoura, Joseph M. Hoolachan, Taylor Scaife, Sara Iqbal, Amarjit Bhomra, Anna J. Kordala, Frederique Scamps, Cedric Raoul, Matthew J. A. Wood, Melissa Bowerman

**Affiliations:** 1 Department of Physiology, Anatomy and Genetics, University of Oxford, Oxford, UK; 2 School of Medicine, Keele University, Staffordshire, UK; 3 Department of Paediatrics, University of Oxford, Oxford, UK; 4 School of Life Sciences, Keele University, Staffordshire, UK; 5 INM, Univ Montpellier, INSERM, Montpellier, France; 6 ALS Reference Center, Univ Montpellier, CHU Montpellier, Montpellier, France; 7 Wolfson Centre for Inherited Neuromuscular Disease, RJAH Orthopaedic Hospital, Oswestry, UK

**Keywords:** Amyotrophic lateral sclerosis, Skeletal muscle, TWEAK, Fn14, Exercise, Sex, Metabolism

## Abstract

**Background:**

Amyotrophic lateral sclerosis (ALS) is a devastating and incurable neurodegenerative disease. Accumulating evidence strongly suggests that intrinsic muscle defects exist and contribute to disease progression, including imbalances in whole-body metabolic homeostasis. We have previously reported that tumour necrosis factor (TNF)-like weak inducer of apoptosis (TWEAK) and fibroblast growth factor inducible 14 (Fn14) are significantly upregulated in skeletal muscle of the *SOD1*^*G93A*^ ALS mouse model. While antagonising TWEAK did not impact survival, we did observe positive effects in skeletal muscle. Given that Fn14 has been proposed as the main effector of the TWEAK/Fn14 activity and that Fn14 can act independently from TWEAK in muscle, we suggest that manipulating Fn14 instead of TWEAK in the *SOD1*^*G93A*^ ALS mice could lead to differential and potentially improved benefits.

**Methods:**

We thus investigated the contribution of Fn14 to disease phenotypes in the *SOD1*^*G93A*^ ALS mice. To do so, Fn14 knockout mice (*Fn14*^*−/−*^) were crossed onto the *SOD1*^*G93A*^ background to generate *SOD1*^*G93A*^*;Fn14*^*−/−*^ mice. Investigations were performed on both unexercised and exercised (rotarod and/or grid test) animals (wild type (WT), *Fn14*^−/−^, *SOD1*^*G93A*^ and *SOD1*^*G93A*^*;Fn14*^*−/−*^).

**Results:**

Here, we firstly confirm that the TWEAK/Fn14 pathway is dysregulated in skeletal muscle of *SOD1*^*G93A*^ mice. We then show that Fn14-depleted *SOD1*^*G93A*^ mice display increased lifespan, myofiber size, neuromuscular junction endplate area as well as altered expression of known molecular effectors of the TWEAK/Fn14 pathway, without an impact on motor function. Importantly, we also observe a complex interaction between exercise (rotarod and grid test), genotype, disease state and sex that influences the overall effects of Fn14 deletion on survival, expression of known molecular effectors of the TWEAK/Fn14 pathway, expression of myosin heavy chain isoforms and myofiber size.

**Conclusions:**

Our study provides further insights on the different roles of the TWEAK/Fn14 pathway in pathological skeletal muscle and how they can be influenced by age, disease, sex and exercise. This is particularly relevant in the ALS field, where combinatorial therapies that include exercise regimens are currently being explored. As such, a better understanding and consideration of the interactions between treatments, muscle metabolism, sex and exercise will be of importance in future studies.

**Supplementary Information:**

The online version contains supplementary material available at 10.1186/s13395-024-00356-0.

## Background

Amyotrophic lateral sclerosis (ALS) is a devastating and currently incurable neurodegenerative disease. Once symptomatic, the median survival of patients is usually between 3 and 5 years. Clinical manifestations typically occur in mid-life, followed by the rapid and progressive wasting of muscles and subsequent paralysis [1]. ALS can be sporadic (~ 80%) or familial (~ 20%) [2], and in the latter case can be caused by numerous genetic mutations with the most common being in *chromosome 9 open reading frame 72* (*C9ORF72*) [3, 4], *superoxide dismutase 1* (*SOD1*) [5], *Fused in Sarcoma* (*FUS*) [6, 7] and *TAR DNA-binding protein 43* (*TDP-43*) [8–10]. Both sporadic and familial ALS patients present similar symptoms and pathophysiology. While the primary pathological target of ALS is undeniably the motor neurons (both upper and lower), accumulating evidence strongly suggests that intrinsic muscle defects exist and contribute to disease progression and presentation [11]. Indeed, the muscle-restricted expression of mutant SOD1 results in a canonical ALS pathophysiology [12, 13]. Furthermore, aberrant genetic, biochemical, developmental, regulatory and physiological changes prior to, or accompanying, motor neuron loss are observed in ALS muscle and progenitor cells [11]. As muscle plays an important role in maintaining systemic energy homeostasis [14], intrinsic muscle defects can have severe consequences on whole-body metabolic homeostasis. Interestingly, instances of insulin resistance [15], hyperlipidemia [16], hyperglycemia [17], aberrant fatty acid metabolism [18], hyperglucagonemia [19], glucose intolerance [18] and development of diabetes [20] have all been reported in ALS patients and animal models. Furthermore both dietary and exercise interventions, which are direct modulators of muscle metabolism [21], have been demonstrated to impact disease progression in ALS patients and animal models [22–24]. Thus, uncovering and targeting pathological molecular effectors in ALS muscle may lead to tissue-specific and whole-body improvements [11, 25, 26].

One important pathway that contributes to skeletal muscle health, function and metabolism is controlled by the binding of the tumour necrosis factor (TNF)-like weak inducer of apoptosis (TWEAK) ligand to the TNF fibroblast growth factor inducible 14 (Fn14) receptor [27, 28]. Interestingly, the TWEAK/Fn14 pathway can impact muscle positively or negatively depending on the levels of TWEAK present. High levels are typically associated with detrimental effects while low levels have a beneficial impact [27, 28]. Similarly, Fn14 expression is typically very low in healthy muscle and becomes upregulated in muscle atrophy conditions, which can lead to sustained muscle pathology if not restored to normal levels [27, 28]. Furthermore, TWEAK and Fn14 have both been implicated in the regulation of key muscle metabolic effectors such as peroxisome proliferative activated receptor, gamma, coactivator 1 alpha (PGC-1α), Slc2a4 solute carrier family 2, member 4 (GLUT4), hexokinase 2 (HKII) and Krüppel-like transcription factor 15 (KLF15) [29].

What still remains unclear however, is the potential role of the TWEAK/Fn14 pathway in neuromuscular conditions, where chronic muscle wasting occurs due to motor neuron loss and muscle denervation [30]. In an attempt to explore this further, we have previously investigated the TWEAK/Fn14 signalling cascade in mouse models of ALS and spinal muscular atrophy (SMA), a childhood neuromuscular disease [31]. In pre-weaned SMA mice, we observed a significant downregulation of *Tweak* and *Fn14* in various skeletal muscles during disease progression, accompanied by the expected dysregulation of *PGC-1α*, *Glut4*, *HKII* and *Klf15* [32]. Interestingly, administering Fc-TWEAK, an agonist of the pathway, to SMA mice, improved several canonical disease phenotypes [32]. Conversely, we have previously observed that *Tweak* and *Fn14* are significantly upregulated in skeletal muscle of *SOD1*^*G93A*^ ALS mice during disease progression [33]. While antagonising TWEAK, either genetically or pharmacologically, did not impact survival, we did observe positive effects in skeletal muscle [33]. Since the receptor has been proposed as the main effector of the TWEAK/Fn14 pathway activity [34] and that Fn14 can act independently from TWEAK in muscle [35], it is possible that manipulating Fn14 instead of TWEAK in the *SOD1*^*G93A*^ ALS mice could lead to differential and/or improved benefits.

In this study, we investigated the effect of Fn14 depletion on disease progression and muscle pathology in *SOD1*^*G93A*^ ALS mice by crossing Fn14 knockout mice (*Fn14*^*−/−*^) with the *SOD1*^*G93A*^ mouse model. We confirmed that the TWEAK/Fn14 pathway is dysregulated in the skeletal muscle of *SOD1*^*G93A*^ mice. We then showed that Fn14-depleted *SOD1*^*G93A*^ mice had an increased lifespan and decreased muscle pathology, which was dependent on exposure to exercise and sex. Our study provides further insights into the different roles of the TWEAK/Fn14 pathway in skeletal muscle and how they may be influenced by age, disease, sex and exercise.

## Methods

### Animals and animal procedures

*SOD1*^*G93A*^ mice (B6.Cg-Tg(SOD1*G93A)1Gur/J) were obtained from Jackson Laboratories (Strain #: 004435). The *Fn14‍*^*−‍/‍−‍*^ mouse model [36] was provided by Biogen. Both strains were on a C57BL/6 genetic background.

Experimental procedures with live animals were authorized and approved by the University of Oxford ethics committee and UK Home Office (Project licenses PDFEDC6F0 and 30/2907) in accordance with the Animals (Scientific Procedures) Act 1986.

For survival studies, mice were weighed and monitored daily and culled upon reaching their defined humane endpoint as specified in the project license.

For all experiments, litters were randomly assigned treatment at birth. Sample sizes were determined based on similar studies with *SOD1*^*G93A*^ mice.

For the grid test, we used our previously described protocol [33], whereby starting with a 40 g metal grid (followed by 30, 20 and 10 g grids), we measured the time (maximum 30 s) the animal held on to the grid before dropping it. The experiment was repeated three times with each grid. Muscle strength (arbitrary units) was quantified with the following formula: (40 g × best time) + (30 g × best time) + (20 g × best time) + (10 g × best time).

For the rotarod test, we followed the previously described protocol [37], whereby mice were placed on the rotarod (opposite orientation to rotation) with an acceleration protocol of 4 to 40 rpm in 300 s. The latency to fall (s) and highest rpm reached was recorded.

To reduce the total number of mice used, the fast-twitch *tibialis anterior* (TA) and gastrocnemius muscles from the same mice were used for molecular and histological analyses, respectively.

### qPCRs

RNA was extracted from tissues with the RNeasy kit (Qiagen) or with a Isolate II RNA Mini Kit (Bioline) as per the manufacturers’ instructions. The same RNA extraction method was employed for similar experiments and equal RNA amounts were used between samples within the same experiments. cDNA was prepared with the High-capacity cDNA Kit (Life Technologies) or qPCRBIO cDNA Synthesis Kit (PBCR Biosystems) according to the manufacturers’ instructions. The same reverse transcription method was employed for similar experiments. The cDNA template was amplified on a StepOnePlus Real-Time PCR Thermocycler (Life Technologies) with SYBR Green Mastermix (Applied Biosystems) or with qPCRBIO SyGreen Blue Mix Hi-ROX (PCR Biosystems). The same amplification method was used for similar experiments. qPCR data was analysed using the StepOne Software v2.3 (Applied Biosystems). Primers used for qPCR were obtained from IDT and sequences for primers were self-designed (Supplementary Table 1). Relative gene expression was quantified using the Pfaffl method [38] and primer efficiencies were calculated with the LinRegPCR software. The relative expression of all genes of interest was normalised to the expression of *RNA polymerase II polypeptide J* (*PolJ*) [39].

### Immunoblots

Freshly prepared RIPA buffer (50 mM Tris pH 8.8, 150 mM NaCl, 1% NP-40, 0.5% sodium deoxycholate, 0.1% sodium dodecyl-sulphate (SDS) and complete mini-proteinase inhibitors (Roche)) was used to homogenize tissue. Equal amounts of total protein were loaded in the wells, as measured by Bradford Assay. Protein samples were first diluted 1:1 with Laemmli sample buffer (Bio-Rad, Hemel Hempstead, UK) containing 5% β-mercaptoethanol (Sigma) and heated at 100 °C for 10 min. Next, samples were loaded on freshly made 1.5 mm 12% polyacrylamide separating and 5% stacking gel and electrophoresis was performed at 120 V for ~ 1.5 h in running buffer. Proteins were then transferred from the gel onto to a polyvinylidene fluoride membrane (Merck Millipore) via electroblotting at 120 V for 60 min in transfer buffer containing 20% methanol. Membranes were then incubated for 2 h in Odyssey Blocking Buffer (Licor). The membrane was then probed overnight at 4 °C with the primary antibodies (p105/p50, Abcam ab32360, 1:1000; Actin, Abcam ab3280, 1:1000) in Odyssey Blocking Buffer and 0.1% Tween-20. The next day, after three 10-min washes in phosphate buffered saline (PBS), the membrane was incubated for 1 h at room temperature with secondary antibodies (goat anti-rabbit IgG 680RD, LI-COR 926–68,071, 1:1000,; goat anti-mouse IgG 800CW, LI-COR, 926–32,210, 1:1000). Lastly, the membrane was washed three times for 10 min in PBS and visualized by scanning the 700 nm and 800 nm channels on the LI-COR Odyssey CLx infrared imaging system (LI-COR) for 2.5 min per channel. The background was subtracted and signal of protein of interest was divided by signal of the housekeeping protein (actin).

### Laminin staining of skeletal muscles

*Tibialis anterior* (TA) muscles were fixed in 4% paraformaldehyde (PFA) overnight. Tissues were sectioned (13 μm) and incubated in blocking buffer (0.3% Triton-X, 20% foetal bovine serum (FBS) and 20% normal goat serum in PBS) for 2 h. After blocking, tissues were stained overnight at 4 °C with rat anti-laminin (Sigma L0663, 1:1000) in blocking buffer. The next day, tissues were washed in PBS and probed using a goat-anti-rat IgG 488 secondary antibody (Invitrogen A-11006, 1:500) for one hour. PBS-washed tissues were mounted in Fluoromount-G (Southern Biotech). Images were taken with a microscope equipped with a 20X objective. Quantitative assays were performed blinded on 3–5 mice for each group and five sections per mouse. Myofiber area was measured using Fiji (ImageJ) [40].

### Endplate staining of skeletal muscles

Endplates were stained as previously described [41]. Briefly, whole TA muscle was harvested and fixed in 4% PFA for 15 min. Muscles were incubated with α-bungarotoxin (α-BTX) conjugated to tetramethylrhodamine (BT00012, Biotium, 1:100) at RT for 30 min with ensuing PBS washes. Finally, 2–3 thin filets per muscle were sliced and mounted in Fluoromount-G (Southern Biotech). Images were taken with a confocal microscope, with a 20X objective. The experimenter quantifying endplate size was blinded to the genotype of the animals until all measurements were finalized.

### Statistical analyses

All statistical analyses were done with the most up to date GraphPad Prism software at time of writing. When appropriate, a Student’s unpaired two-tail *t*-test, a one-way analysis of variance (ANOVA) or a two-way ANOVA was used. Post-hoc analyses used are specified in Figure Legends. Outliers were identified via the Grubbs’ test. For the Kaplan–Meier survival analysis, the log-rank test was used and survival curves were considered significantly different at *p* < 0.05.

## Results

### The Fn14 signalling cascade is dysregulated in skeletal muscle of *SOD1*^*G93A*^ mice during disease progression

**Fig. 1:**
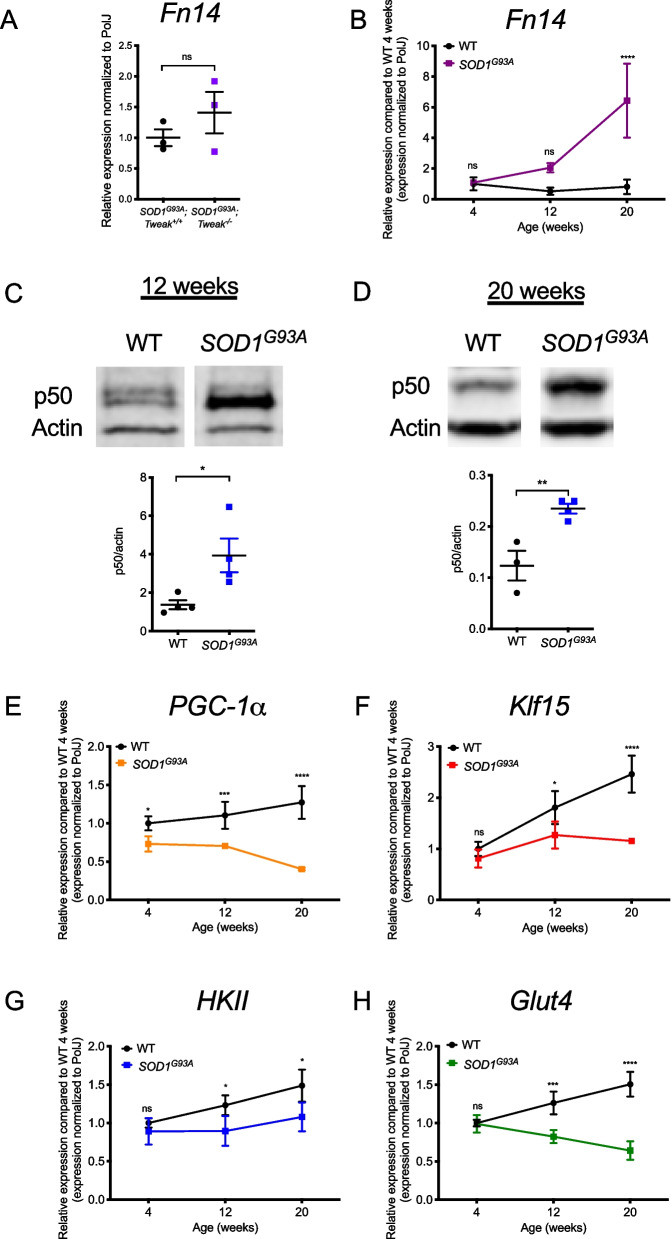
Aberrant expression of the TWEAK/Fn14 signaling pathway in skeletal muscle of *SOD1*^*G93A*^ ALS mice. **A** qPCR analysis of *Fn14* mRNA expression in gastrocnemius muscle from 20-week-old *SOD1*^*G93A*^*;Tweak*^+*/*+^ and *SOD1*^*G93A*^*;Tweak*^*−/−*^ males. Normalized relative expressions are compared to *SOD1*^*G93A*^*;Tweak*^+*/*+^. Data are scatter dot plot mean ± SEM, *n* = 3 animals per genotype, unpaired *t test*; ns, not significant. **B** qPCR analysis of *Fn14* mRNA expression in the *tibialis anterior* (TA) of *SOD1*^*G93A*^ and wild type (WT) mice at 4 (pre-symptomatic), 12 (early symptomatic) and 20 (late symptomatic) weeks. Normalized relative expressions are compared to WT 4 weeks. Data are mean ± SEM, *n* = 3–4 animals per experimental group, two-way ANOVA, ****p < 0.0001. **C**-**D** Quantification of NF-κB p50/actin protein levels in the TAs of 12- (**C**) and 20-week-old (**D**) *SOD1*^*G93A*^ and WT. Images are representative immunoblots. Data are scatter dot plot mean ± SEM, *n* = 3–4 animals per experimental group, unpaired *t test*, *p* = 0.0302 (12 weeks), *p* = 0.0088 (20 weeks). **E**–**F** qPCR analysis of *PGC-1α* (**E**), *Klf15* (**F**), *HKII* (**G**) and *Glut4* (**H**) mRNAs in TAs of 4-, 12- and 20-week-old *SOD1*.^*G93A*^ and WT. Normalized relative expressions are compared to WT 4 weeks. Data are mean ± SEM, *n* = 3–4 animals per experimental group, two-way ANOVA, *p < 0.05, ***p < 0.001

We firstly compared Fn14 expression in the skeletal muscle of 20-week-old symptomatic *SOD1*^*G93A*^ and *SOD1*^*G93A*^*;Tweak*^*−/−*^ mice, which showed no significant difference in *Fn14* mRNA expression (Fig. 1A). This suggests that genetically depleting the ligand (TWEAK) was not sufficient to reduce the expression of the receptor (Fn14). Since Fn14 is a key factor in modulating the activity of the TWEAK/Fn14 pathway [34], its persistent expression despite Tweak depletion may have limited the benefits on muscle pathology and disease progression.

We thus set out to further characterize the Fn14 signalling cascade in skeletal muscle of *SOD1*^*G93A*^ males. We started by reproducing our previously published data [33] and demonstrated that *Fn14* mRNA levels in the *tibialis anterior* (TA) of *SOD1*^*G93A*^ and wild type (WT) mice are similar in 4- (pre-symptomatic) and 12-week-old (early symptomatic) animals, while there is a significant increase in 20-week-old (late symptomatic) *SOD1*^*G93A*^ mice (Fig. 1B). We next assessed the expression of nuclear factor kappa-light-chain-enhancer of activated B cells (NF-κB) subunit p50, a direct downstream effector of TWEAK/Fn14 signalling in skeletal muscle [28, 42] that mediates pathological events in muscle when chronically activated [43]. We found that the expression of NF-κB subunit p50 was significantly upregulated in the TAs of *SOD1*^*G93A*^ mice at both early symptomatic (Fig. 1C) and late symptomatic (Fig. 1D) time-points compared to WT animals, supporting an increased activity of TWEAK/Fn14 activity in skeletal muscle of ALS mice. Next, we evaluated the gene expression of *PGC-1α*, *Klf15*, *HKII* and *Glut4*. Interestingly, we observed a significant decrease in the expression of *PGC-1α* (Fig. 1E), *Klf15* (Fig. 1F), *HKII* (Fig. 1G) and *Glut4* (Fig. 1H) in the TA muscles of 12- and 20-week-old *SOD1*^*G93A*^ mice compared to WT animals, providing further support for increased Fn14 expression in *SOD1*^*G93A*^ mice.

Together, our results demonstrate an aberrant hyperactivity of TWEAK/Fn14 signalling in the skeletal muscle of *SOD1*^*G93A*^ mice, impacting key regulatory downstream effectors known to influence overall skeletal muscle health and metabolic homeostasis.

### Genetic deletion of Fn14 increases survival of *SOD1*^*G93A*^ mice

**Fig. 2:**
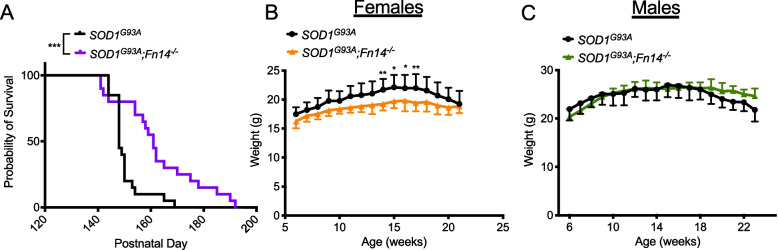
Genetic deletion of *Fn14* increases survival of *SOD1*^*G93A*^ ALS mice. **A** Survival curves of untreated *SOD1*^*G93A*^ and *SOD1*^*G93A*^*;Fn14*^*−/−*^ mice (males and females combined). Data are represented as Kaplan–Meier survival curves, *n* = 20 animals per experimental group, Log-rank (Mantel-Cox), *p* = 0009. **B**-**C** Weekly weights of *SOD1*^*G93A*^ and *SOD1*^*G93A*^*;Fn14*.^*−/−*^ females (**B**) and males (**C**) from 6 weeks to humane endpoint. Data are mean ± SEM, *n* = 9–11 animals per experimental group, two-way ANOVA, *p < 0.05, **p < 0.01

We sought to determine if decreasing TWEAK/Fn14 activity in *SOD1*^*G93A*^ mice would improve muscle health and slow disease progression. As described above, we have previously modulated TWEAK activity both genetically and pharmacologically [33]. We thus decided to investigate the impact of depleting the activity of the receptor to abolish downstream signalling effector of the TWEAK/Fn14 pathway [34]. We crossed *SOD1*^*G93A*^ mice with *Fn14*^*−/−*^ mice [36], to generate ALS mice with a homozygous deletion of Fn14. Interestingly, we found that *SOD1*^*G93A*^*;Fn14*^*−/−*^ mice had a significantly increased lifespan compared to *SOD1*^*G93A*^ mice (females and males combined) (Fig. 2A) without any substantial improvements in weight (Fig. 2B, C). In fact, *SOD1*^*G93A*^*;Fn14*^*−/−*^ females tended to weigh less than *SOD1*^*G93A*^ females (Fig. 2B), while there were no significant differences between *SOD1*^*G93A*^*;Fn14*^*−/−*^ and *SOD1*^*G93A*^ males (Fig. 2C). Nevertheless, Fn14 depletion appears to have an overall positive impact on disease progression in *SOD1*^*G93A*^ mice.

### Genetic deletion of Fn14 improves muscle pathology in *SOD1*^*G93A*^ mice

**Fig. 3:**
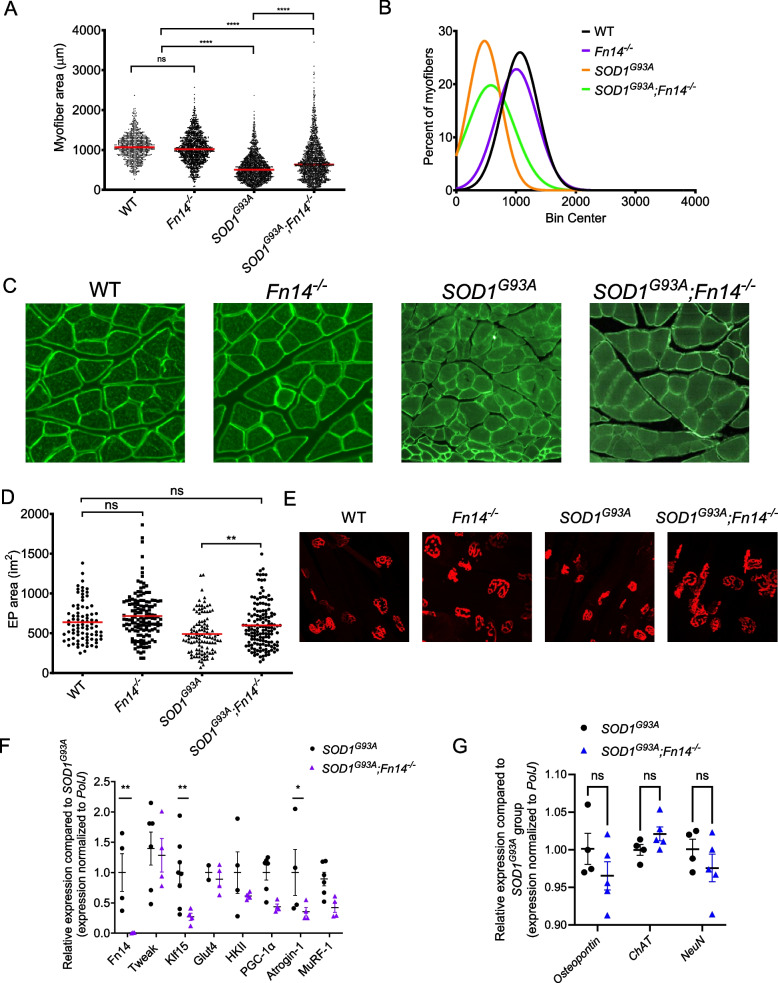
Genetic deletion of *Fn14* improves muscle phenotypes in *SOD1*^*G93A*^ ALS mice. **A** Quantification of myofiber area of laminin-stained cross-sections of gastrocnemius muscles from 20-week-old WT, *Fn14*^*−/−*^, *SOD1*^*G93A*^ and *SOD1*^*G93A*^*;Fn14*^*−/−*^ males. Data are dot plot and mean, *n* = 3–4 animals per experimental group (> 800 myofibers per experimental group), one-way ANOVA, ns = not significant, ****p < 0.0001. **B** Relative frequency distribution of myofiber size in gastrocnemius muscles from 20-week-old WT, *Fn14*^*−/−*^, *SOD1*^*G93A*^ and *SOD1*^*G93A*^*;Fn14*^*−/−*^ mice. **C** Representative images of laminin-stained cross-sections of gastrocnemius muscles from 20-week-old WT, *Fn14*^*−/−*^, *SOD1*^*G93A*^ and *SOD1*^*G93A*^*;Fn14*^*−/−*^ mice. **D** Quantification of neuromuscular junction endplate (EP) area of alpha-bungarotoxin-stained TA muscles from 20-week-old WT, *Fn14*^*−/−*^, *SOD1*^*G93A*^ and *SOD1*^*G93A*^*;Fn14*^*−/−*^ mice. Data are dot plot and mean, *n* = 3–4 animals per experimental group (> 80 myofibers per experimental group), one-way ANOVA, ns = not significant, **p < 0.01. **E** Representative images of alpha-bungarotoxin-stained TA muscles from 20-week-old WT, *Fn14*^*−/−*^, *SOD1*^*G93A*^ and *SOD1*^*G93A*^*;Fn14*^*−/−*^ mice. **F** qPCR analysis of *Fn14*, *Tweak*, *Klf15*, *Glut4*, *HKII*, *PGC-1α*, *Atrogin-1* and *MuRF-1* mRNA in TA muscles from *SOD1*^*G93A*^ and *SOD1*^*G93A*^*;Fn14*^*−/−*^ mice. Normalized relative expressions are compared to *SOD1*^*G93A*^ for each gene. Data are scatter dot plot mean ± SEM, *n* = 3–8 animals per experimental group, two-way ANOVA, *p < 0.05. **G** qPCR analysis of *osteopontin*, *ChAT* and *NeuN* in spinal cord from *SOD1*^*G93A*^ and *SOD1*^*G93A*^*;Fn14*^*−/−*^ mice. Normalized relative expressions are compared to *SOD1*.^*G93A*^ for each gene. Data are scatter dot plot mean ± SEM, *n* = 4–5 animals per experimental group, two-way ANOVA, *p < 0.05

We next determined the impact of Fn14 depletion on previously characterised skeletal muscle pathologies in 20-week-old *SOD1*^*G93A*^ males. We firstly measured the myofiber area in the gastrocnemius muscle of WT, *Fn14*^*−/−*^, *SOD1*^*G93A*^ and *SOD1*^*G93A*^*;Fn14*^*−/−*^ mice as muscle wasting is evident in these ALS mice at that symptomatic time-point [33]. We found that while Fn14 depletion in WT animals had no impact on myofiber size, there was a significant increase in myofiber size in *SOD1*^*G93A*^*;Fn14*^*−/−*^ mice compared to *SOD1*^*G93A*^ animals (Fig. 3A-C).

We also investigated the impact of Fn14 deletion on post-synaptic neuromuscular junction (NMJ) pathologies by evaluating endplate size, which is typically reduced in ALS mice [44] and associated with muscle size [45]. Similar to myofiber size, we observed that Fn14 depletion did not influence the NMJ endplate size in the TA muscles of WT animals, but it significantly increased endplate size in *SOD1*^*G93A*^ mice (Fig. 3D, E).

To determine the impact of Fn14 deletion on skeletal muscle at a molecular level, we investigated the gene expression of molecular effectors associated with the TWEAK/Fn14 signalling cascade (*Fn14*, *Tweak*, *Klf15*, *Glut4*, *HKII* and *PGC-1*α) [29] and muscle atrophy markers (*Atrogin-1* and *MuRF-1*) [46]. We found that the complete elimination of Fn14 in TA muscles of *SOD1*^*G93A*^ mice did not influence the expression of *Tweak*, *Glut4*, *HKII*, *PGC-1*α and *MuRF-1* (Fig. 3F). However, we observed a significant decrease in the expression of *Klf15* and, importantly, the atrogene *Atrogin-1* (Fig. 3F).

Finally, given the effects observed in skeletal muscle and at the NMJ, we evaluated the effect of systemic Fn14 deletion on the expression of motor neuron markers (osteopontin [47, 48], choline acetyltransferase (ChAT) [49], and neuronal nuclear antigen (NeuN) [50]) in the spinal cord. We observed that the levels of *osteopontin*, *ChAT* and *NeuN* were not significantly different between *SOD1*^*G93A*^ and *SOD1*^*G93A*^*;Fn14*^*−/−*^ mice (Fig. 3G), implying that the origin of the benefits of Fn14 depletion on the NMJ in *SOD1*^*G93A*^ animals is most likely the muscle and not the spinal cord.

Combined, our analyses of symptomatic mice reveal that deletion of *Fn14* in *SOD1*^*G93A*^ mice improves several muscle wasting phenotypes without impacting the expression of spinal cord motor neuron markers. This suggests that the aberrant increased expression of Fn14 in skeletal muscle of *SOD1*^*G93A*^ animals may contribute to the muscle pathologies that characterise the disease.

### Enhanced physical activity and Fn14 depletion both have positive effects on survival of *SOD1*^*G93A*^ mice

**Fig. 4:**
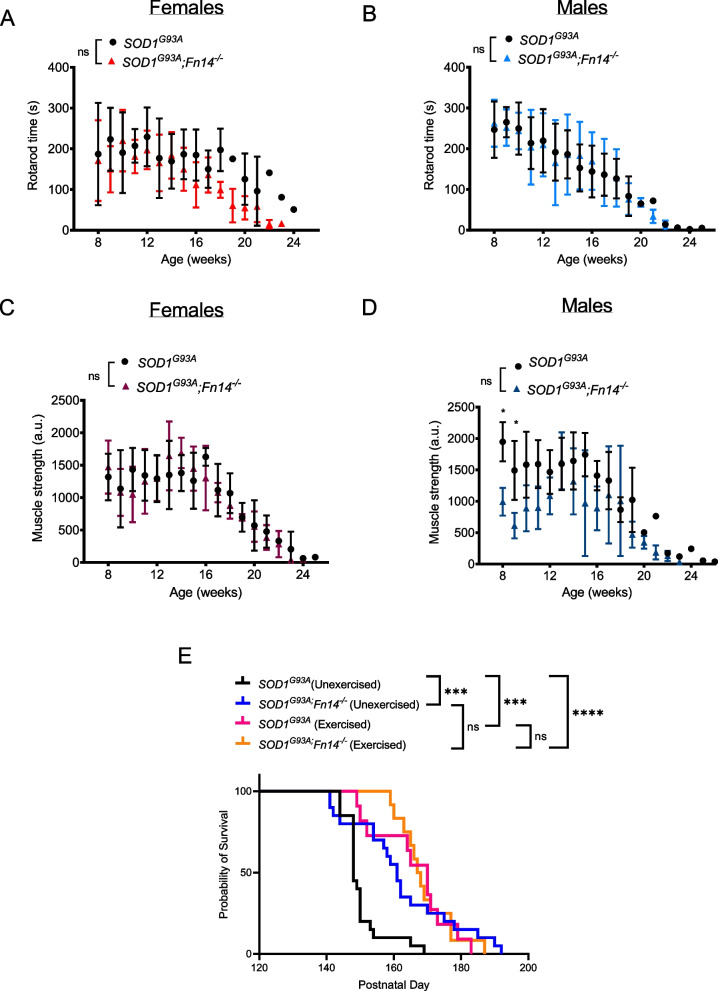
Weekly exercise tests do not reveal any improvements in motor function of Fn14-depleted *SOD1*^*G93A*^ mice and induce benefits on lifespan independent of Fn14 depletion. *SOD1*^*G93A*^ and *SOD1*^*G93A*^*;Fn14*^*−/−*^ mice performed both the rotarod and grid test weekly from 8 weeks to humane endpoint. **A-B**) Time in seconds (s) spent on rotarod before falling (maximum 300 s) for *SOD1*^*G93A*^ and *SOD1*^*G93A*^*;Fn14*^*−/−*^ female (**A**) and male (**B**) mice. Data are mean ± SEM, *n* = 5–7 animals per experimental group, two-way ANOVA, ns = not significant. **C-D**) Muscle strength (arbitrary units (a.u.) for *SOD1*^*G93A*^ and *SOD1*^*G93A*^*;Fn14*^*−/−*^ female (**C**) and male (**D**) mice. Data are mean ± SEM, *n* = 5–7 animals per experimental group, two-way ANOVA, ns = not significant, *p < 0.05. **E**) Survival curves of *SOD1*^*G93A*^ and *SOD1*^*G93A*^*;Fn14*.^*−/−*^ mice that performed both the rotarod and grid test weekly from 8 weeks to humane endpoint (males and females combined). Data are represented as Kaplan–Meier survival curves, *n* = 11–12 animals per experimental group, Log-rank (Mantel-Cox), ns = not significant, ***p < 0.001, ****p < 0.0001

We assessed if the observed molecular and histological benefits in the muscles of Fn14-depleted ALS mice translated into improved motor performance. *SOD1*^*G93A*^ and *SOD1*^*G93A*^*;Fn14*^*−/−*^ mice therefore performed a weekly rotarod [51] and grid test [33, 52], starting at 8 weeks and ending when the animals reached their defined humane endpoint. Both tests have previously been used in *SOD1*^*G93A*^ mice [33, 53] and are aimed at evaluating motor balance and coordination (rotarod) and strength (grid test). We found that there were no significant differences in the time spent on the rotarod between *SOD1*^*G93A*^ and *SOD1*^*G93A*^*;Fn14*^*−/−*^ female and male mice (Fig. 4A, B). With the grid test, no significant difference in muscle strength was observed between *SOD1*^*G93A*^ and *SOD1*^*G93A*^*;Fn14*^*−/−*^ females (Fig. 4C), while *SOD1*^*G93A*^ males were significantly stronger than *SOD1*^*G93A*^*;Fn14*^*−/−*^ males at the very early pre-symptomatic time-points (Fig. 4D). Although these results suggest that Fn14 depletion does not enhance muscle strength and/or performance in *SOD1*^*G93A*^ mice, this might be due to the independent benefits provided by the weekly rotarod and grid tests exercises. Indeed, exercised *SOD1*^*G93A*^ animals had a significantly greater lifespan than unexercised *SOD1*^*G93A*^ mice (Fig. 4E). As such, exercised *SOD1*^*G93A*^ and *SOD1*^*G93A*^*;Fn14*^*−/−*^ mice had similar survivals, suggesting that both exercise and Fn14 depletion can improve survival in *SOD1*^*G93A*^ mice. While the median lifespan of exercised *SOD1*^*G93A*^ and *SOD1*^*G93A*^*;Fn14*^*−/−*^ mice were not significantly different, there did appear to be a delay in the early deaths in the exercised *SOD1*^*G93A*^*;Fn14*^*−/−*^ group, pointing towards a potential combination of independent and dependent mechanisms. Of note, there was also no significant difference between the survival of heterozygous *SOD1*^*G93A*^*;Fn14*^*+/−*^ and homozygous *SOD1*^*G93A*^*;Fn14*^*−/−*^ mice that underwent weekly rotarod and grid test assessments (Supplementary Fig. 1).

### Fn14 depletion changes molecular response of *SOD1*^*G93A*^ muscle to exercise

To further elucidate the potential complex interactions between exercise, disease state and Fn14 depletion, 12-week-old mice underwent the rotarod or grid test for 5 consecutive days. The 12-week time point was chosen as it is an early symptomatic age for *SOD1*^*G93A*^ mice that still allows them to complete both exercise regimens to the same extent as WT and *Fn14*^*−/−*^ animals. The TAs were harvested 2 h after the last test and compared to those of unexercised sex- and age-matched mice for the expression of the known TWEAK/Fn14 molecular effectors and atrogenes investigated above.

**Fig. 5:**
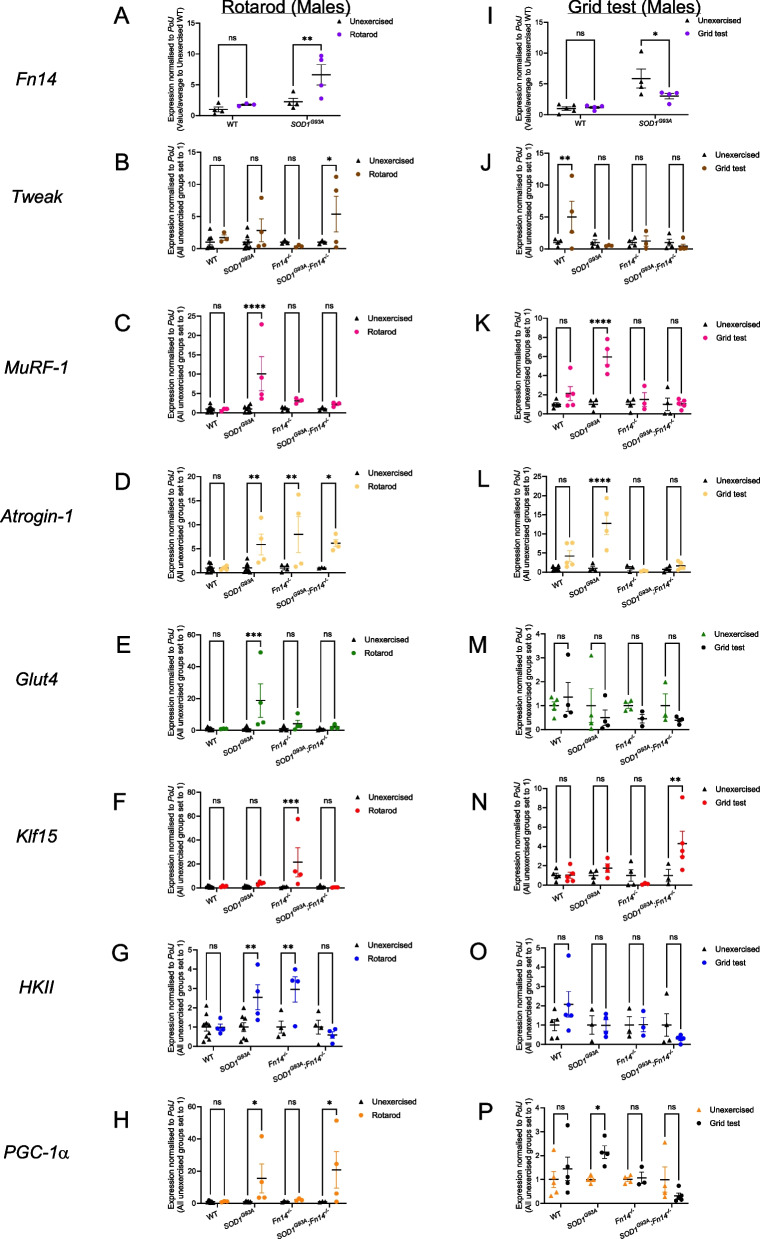
Type of exercise and genotype impact the expression of *Tweak*, *Fn14* and their downstream effectors in male mice. 12-week-old WT, *Fn14*^*−/−*^, *SOD1*^*G93A*^ and *SOD1*^*G93A*^*;Fn14*^*−/−*^ males were either placed on the rotarod (A-H) or performed the grid test (I-P) daily for 5 consecutive days. *Tibialis anterior* (TA) muscles were harvested approximately 2 h after the last bout of exercise. **A** qPCR analysis of *Fn14* mRNA expression in unexercised and rotarod-exercised WT and *SOD1*^*G93A*^ mice. Data are scatter dot plot mean ± SEM, *n* = 3–4 animals per experimental group, two-way ANOVA, ns = not significant, **p < 0.01. **B**-**H** qPCR analysis of *Tweak* (**B**), *MuRF-1* (**C**), *Atrogin-1* (**D**), *Glut4* (**E**), *Klf15* (**F**), *HKII* (**G**), *PGC-1α* (**H**) mRNA expression in unexercised and rotarod-exercised WT, *Fn14*^*−/−*^, *SOD1*^*G93A*^ and *SOD1*^*G93A*^*;Fn14*^*−/−*^ mice. Data are scatter dot plot mean ± SEM, *n* = 4–9 animals per experimental group, two-way ANOVA, ns = not significant, *p < 0.05, ***p < 0.001, ****p < 0.0001. **I** qPCR analysis of *Fn14* mRNA expression in unexercised and grid test-exercised WT and *SOD1*^*G93A*^ mice. Data are scatter dot plot mean ± SEM, *n* = 3–4 animals per experimental group, two-way ANOVA, ns = not significant, **p < 0.01. **J**-**P** qPCR analysis of *Tweak* (**J**), *MuRF-1* (**K**), *Atrogin-1* (**L**), *Glut4* (**M**), Klf15 (**N**), *HKII* (**O**), *PGC-1α* (**P**) mRNA expression in unexercised and grid test-exercised WT, *Fn14*^*−/−*^, *SOD1*^*G93A*^ and *SOD1*^*G93A*^*;Fn14*.^*−/−*^ mice. Data are scatter dot plot mean ± SEM, n = 4–9 animals per experimental group, two-way ANOVA, ns = not significant, *p < 0.05, ***p < 0.001, ****p < 0.0001

First, we assessed and compared TAs from unexercised and rotarod-trained males. Interestingly, we observed that *Fn14* expression was significantly upregulated in rotarod-trained *SOD1*^*G93A*^ mice compared to unexercised *SOD1*^*G93A*^ animals, while *Fn14* levels remained unchanged in WT animals (Fig. 5A), suggesting a yet to be determined role for Fn14 in exercised *SOD1*^*G93A*^ muscle. Next, we compared the expression of *Tweak*, *MuRF-1*, *Atrogin-1*, *Glut4*, *Klf15*, *HKII* and *PGC-1*α in unexercised and rotarod-trained WT, *Fn14*^*−/−*^, *SOD1*^*G93A*^ and *SOD1*^*G93A*^*;Fn14*^*−/−*^ mice. We found that *Tweak* was significantly increased only in rotarod-trained *SOD1*^*G93A*^*;Fn14*^*−/−*^ animals compared to unexercised mice (Fig. 5B), suggesting a compensatory mechanism that is plausibly due to reduced levels of its ligand and exercise. The atrogene *MuRF-1* was significantly increased only in the muscles of rotarod-trained *SOD1*^*G93A*^ mice compared to unexercised animals (Fig. 5C), indicating that depletion of Fn14 prevents exercise-induced *MuRF-1* upregulation. However, this effect appears to be specific to *MuRF-1* as *Atrogin-1*, which is significantly upregulated in rotarod-trained *SOD1*^*G93A*^ mice, was also increased in rotarod-trained *Fn14*^*−/−*^ and *SOD1*^*G93A*^*;Fn14*^*−/−*^ mice compared to unexercised cohorts (Fig. 5D). Similarly, the expression of *Glut4* was significantly increased only in rotarod-trained *SOD1*^*G93A*^ mice compared to unexercised animals and remained unchanged in Fn14-depleted groups (Fig. 5E). Interestingly, the expression of *Klf15* was significantly upregulated only in rotarod-trained *Fn14*^*−/−*^ animals compared to unexercised mice (Fig. 5F). As for the expression of *HKII*, it was significantly increased in rotarod-trained *SOD1*^*G93A*^ and *Fn14*^*−/−*^ mice, while it remained unchanged in rotarod-trained WT mice and *SOD1*^*G93A*^*;Fn14*^*−/−*^ compared to unexercised groups (Fig. 5G). Finally, the expression of *PGC-1*α was significantly upregulated only in rotarod-trained *SOD1*^*G93A*^ and *SOD1*^*G93A*^*;Fn14*^*−/−*^ animals compared to unexercised mice (Fig. 5H).

Next, we performed the same investigations in TAs from unexercised and grid test-trained males. Contrary to what was observed in rotarod-trained *SOD1*^*G93A*^ males (Fig. 5A), we found that grid test-trained *SOD1*^*G93A*^ mice expressed significantly less *Fn14* than unexercised *SOD1*^*G93A*^ animals (Fig. 5I), suggesting a distinct response between rotarod and grid test activities. On the other hand, *Tweak* expression was significantly increased only in grid test-trained WT animals compared to unexercised mice, while it remained unchanged in grid test-trained animals of the same genotype (Fig. 5J). The expression of both atrogenes, *MuRF-1* and *Atrogin-1*, was significantly upregulated only in grid-test trained *SOD1*^*G93A*^ mice compared to unexercised animals and restored to low levels when *Fn14* was depleted (Fig. 5K, L). *Glut4* levels were unchanged in all experimental groups when comparing unexercised animals to grid test-trained mice (Fig. 5M). Interestingly, *Klf15* expression was significantly upregulated only in *SOD1*^*G93A*^*;Fn14*^*−/−*^ animals compared to unexercised mice, as it remained unchanged in all other groups (Fig. 5N). Similar to *Glut4*, *HKII* levels were also unchanged in all experimental groups when comparing unexercised animals to grid test-trained mice (Fig. 5O). Finally, *PGC-1α* expression was significantly increased only in grid test-trained *SOD1*^*G93A*^ mice compared to unexercised animals and returned to lower levels in Fn14-depleted animals (Fig. 5P).

**Fig. 6:**
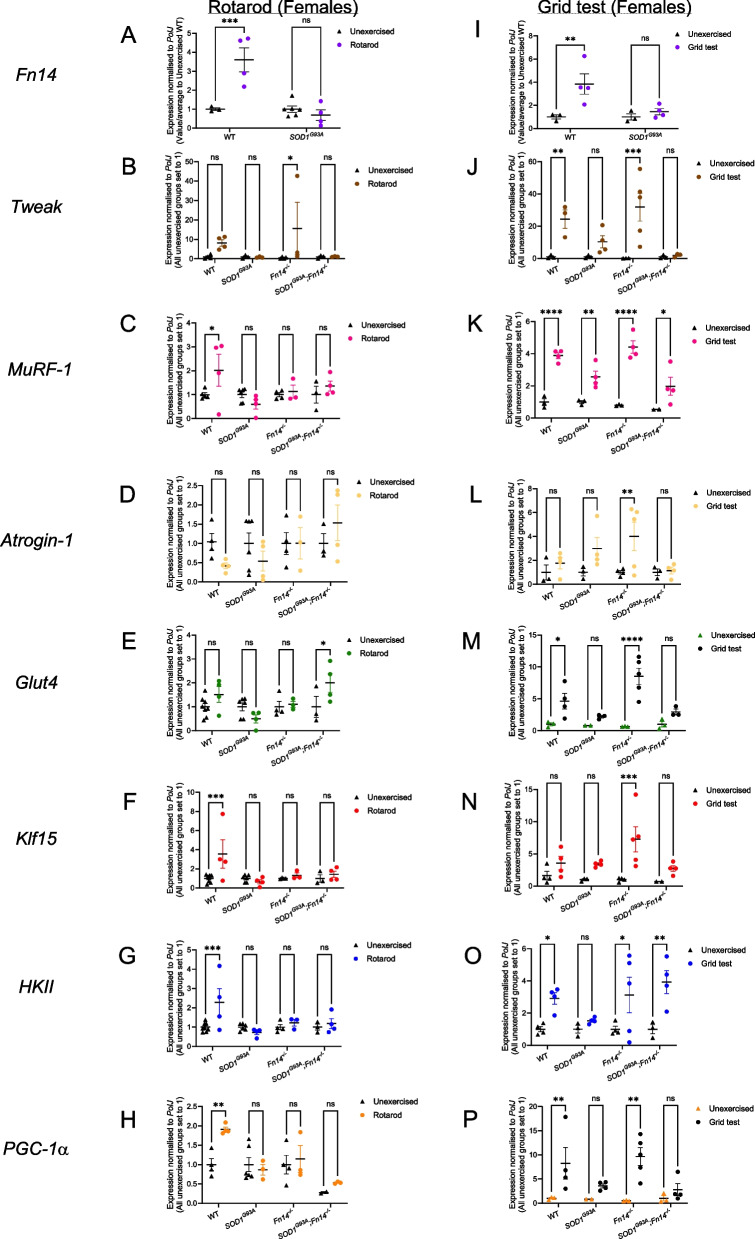
Type of exercise and genotype impact the expression of *Tweak*, *Fn14* and their downstream effectors in female mice. 12-week-old WT, *Fn14*^*−/−*^, *SOD1*^*G93A*^ and *SOD1*^*G93A*^*;Fn14*^*−/−*^ females were either placed on the rotarod (A-H) or performed the grid test (I-P) daily for 5 consecutive days. *Tibialis anterior* (TA) muscles were harvested approximately 2 h after the last bout of exercise. **A** qPCR analysis of *Fn14* mRNA expression in unexercised and rotarod-exercised WT and *SOD1*^*G93A*^ mice. Data are scatter dot plot mean ± SEM, *n* = 4–6 animals per experimental group, two-way ANOVA, ns = not significant, ***p < 0.001. **B-H** qPCR analysis of *Tweak* (**B**), *MuRF-1* (**C**), *Atrogin-1* (**D**), *Glut4* (**E**), *Klf15* (**F**), *HKII* (**G**), *PGC-1α* (**H**) mRNA expression in unexercised and rotarod-exercised WT, *Fn14*^*−/−*^, *SOD1*^*G93A*^ and *SOD1*^*G93A*^*;Fn14*^*−/−*^ mice. Data are scatter dot plot mean ± SEM, *n* = 3–8 animals per experimental group, two-way ANOVA, ns = not significant, *p < 0.05, **p < 0.01, ***p < 0.001. **I**) qPCR analysis of *Fn14* mRNA expression in unexercised and grid test-exercised WT and *SOD1*^*G93A*^ mice. Data are scatter dot plot mean ± SEM, *n* = 3–4 animals per experimental group, two-way ANOVA, ns = not significant, **p < 0.01. **J-P** qPCR analysis of *Tweak* (**J**), *MuRF-1* (**K**), *Atrogin-1* (**L**), *Glut4* (**M**), Klf15 (**N**), *HKII* (**O**), *PGC-1α* (**P**) mRNA expression in unexercised and grid test-exercised WT, *Fn14*^*−/−*^, *SOD1*^*G93A*^ and *SOD1*^*G93A*^*;Fn14*^*−/−*^ mice. Data are scatter dot plot mean ± SEM, *n* = 3–5 animals per experimental group, two-way ANOVA, ns = not significant, *p < 0.05, **p < 0.01, ***p < 0.001, ****p < 0.0001

The same analyses were then performed in females to see if sex was an additional factor influencing the interactions between exercise, disease state and Fn14 depletion. Indeed, in the rotarod female experimental groups, we found that *Fn14* expression was significantly elevated in rotarod-trained WT mice compared to unexercised WT animals, while remaining unchanged in *SOD1*^*G93A*^ mice (Fig. 6A). *Tweak* levels were significantly upregulated only in rotarod-trained *Fn14*^*−/−*^ animals compared to unexercised mice (Fig. 6B). As for the atrogenes, we observed a significant increased expression of *MuRF-1* only in rotarod-trained WT animals compared to unexercised mice (Fig. 6C) and no significant changes in any experimental groups for *Atrogin-1* (Fig. 6D). *Glut4* levels were significantly elevated only in rotarod-trained *SOD1*^*G93A*^*;Fn14*^*−/−*^ animals compared to unexercised mice (Fig. 6E) while the expression of *Klf15*, *HKII* and *PGC-1α* expression were significantly increased only in rotarod-trained WT mice compared to unexercised animals (Fig. 6F-H).

When performing the same comparisons in grid test female experimental groups, we observed a significant increased expression of *Fn14* only in grid test-trained WT animals compared to unexercised mice (Fig. 6I). *Tweak* levels were significantly elevated in both grid test-trained WT and *Fn14*^*−/−*^ mice compared to unexercised animals (Fig. 6J). Interestingly, *MuRF-1* expression was significantly increased in all grid test-trained groups compared to untrained animals (Fig. 6K) while *Atrogin-1* levels were significantly elevated only in grid test-trained *Fn14*^*−/−*^ mice compared to unexercised animals (Fig. 6L). *Glut4* expression was significantly increased in both grid test-trained WT and *Fn14*^*−/−*^ mice compared to unexercised animals (Fig. 6M). *Klf15* levels were significantly elevated only in grid test-trained *Fn14*^*−/−*^ animals compared to unexercised mice (Fig. 6N) while *HKII* expression was significantly increased in all grid test-trained experimental groups compared to unexercised animals except for *SOD1*^*G93A*^ mice where the levels remained unchanged (Fig. 6O). Finally, similar to *Glut4*, *PGC-1α* levels were significantly elevated in both grid test-trained WT and *Fn14*^*−/−*^ mice compared to unexercised animals (Fig. 6P).

Our overall results suggest that exercise regimens have a differential impact on the skeletal muscle of our 12-week-old experimental cohorts, pointing towards specific interactions between sex, genotype, Fn14 depletion and exercise (summarised in Table 1).

**Table 1:**
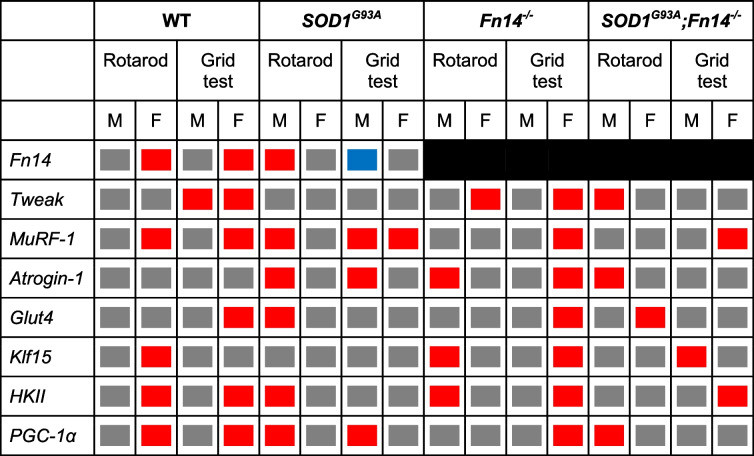
Effect of exercise, sex and genotype on expression of the TWEAK/Fn14 signalling pathway and atrogenes

Red = upregulated

Blue = downregulated

Grey = no change

Black = not applicable

M = Males

F = Females

### Fn14 depletion influences the impact of exercise on expression of myosin heavy chain isoforms

The physiological and biochemical properties of skeletal muscle are, in part, conferred by myosin heavy chain (MyHC) isoforms. Of those, MyHC isoforms IIa, IIx and IIb are specific to fast twitch type of muscles, have been demonstrated to co-exist in the same muscle and their expression can be influenced by exercise [54, 55]. Given that the TA is considered a fast twitch skeletal muscle, we investigated the impact of rotarod and grid test exercise regimens on the gene expression of MyHC IIa, IIx and IIb isoforms in 12-week-old WT, *Fn14*^*−/−*^, *SOD1*^*G93A*^ and *SOD1*^*G93A*^*;Fn14*^*−/−*^ males and females.

**Fig. 7:**
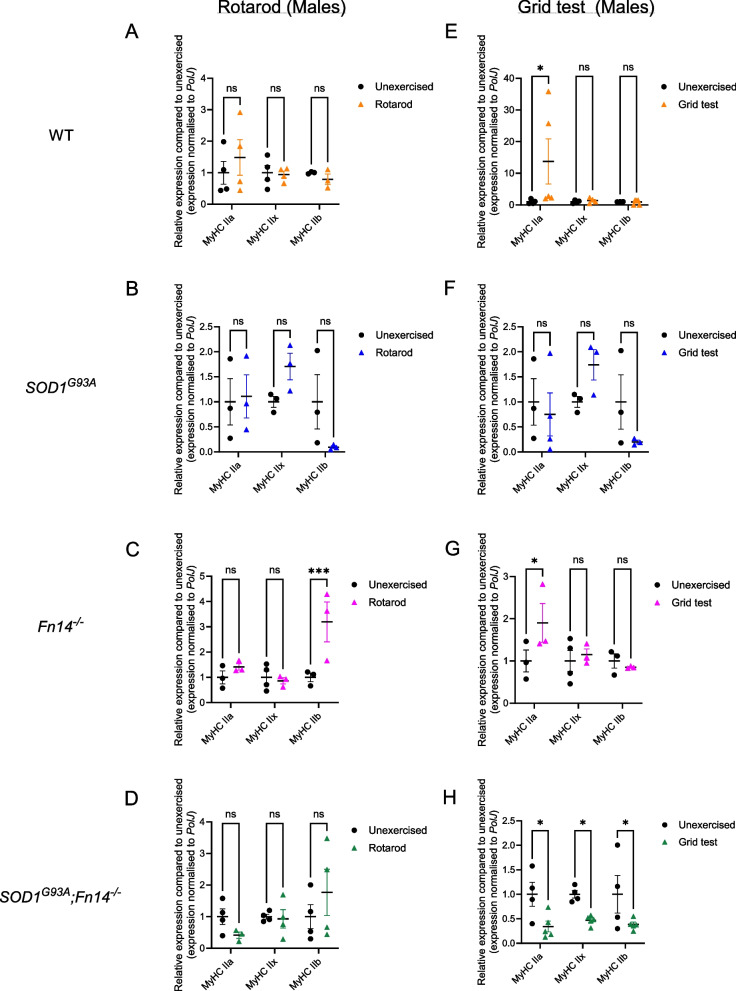
Type of exercise and genotype impact the expression of *MyHC* isoforms *IIa*, *IIx* and *IIb* in male mice. 12-week-old WT, *Fn14*^*−/−*^, *SOD1*^*G93A*^ and *SOD1*^*G93A*^*;Fn14*^*−/−*^ males were either placed on the rotarod (A-D) or performed the grid test (E–H) daily for 5 consecutive days. *Tibialis anterior* (TA) muscles were harvested approximately 2 h after the last bout of exercise. **A-D** qPCR analysis of *MyHC IIa*, *IIx* and *IIb* mRNA expression in unexercised and rotarod-exercised WT (**A**), *SOD1*^*G93A*^ (**B**), *Fn14*^*−/−*^ (**C**) and *SOD1*^*G93A*^*;Fn14*^*−/−*^ (**D**) males. Data are scatter dot plot mean ± SEM, *n* = 3–4 animals per experimental group, two-way ANOVA, ns = not significant, ***p < 0.001. **E–H** qPCR analysis of *MyHC IIa*, *IIx* and *IIb* mRNA expression in unexercised and grid test-exercised WT (**E**), *SOD1*^*G93A*^ (**F**), *Fn14*^*−/−*^ (**G**) and *SOD1*^*G93A*^*;Fn14*.^*−/−*^ (**H**) males. Data are scatter dot plot mean ± SEM, *n* = 3–5 animals per experimental group, two-way ANOVA, ns = not significant, *p < 0.05

In rotarod-trained males, we found that the expression of all three *MyHC* isoforms remained unchanged in WT and *SOD1*^*G93A*^ animals (Fig. 7A, B). Interestingly, we observed a significant increased expression of *MyHC IIb* in rotarod-trained *Fn14*^*−/−*^ mice compared to unexercised animals (Fig. 7C) while the levels of all three *MyHC* isoforms were similar between rotarod-trained and unexercised *SOD1*^*G93A*^*;Fn14*^*−/−*^ mice (Fig. 7D).

In grid test-trained males, only *MyHC IIa* expression was significantly increased in WT animals (Fig. 7E). Similar to the rotarod, the levels of all three *MyHC* isoforms remained unchanged in grid test-trained *SOD1*^*G93A*^ mice (Fig. 7F). Similar to WT animals, only *MyHC IIa* expression was significantly upregulated in grid test-trained *Fn14*^*−/−*^ mice compared to unexercised animals (Fig. 7G). In contrast, the expression of all three *MyHC* isoforms was significantly downregulated in grid test-trained *SOD1*^*G93A*^*;Fn14*^*−/−*^ animals compared to unexercised mice (Fig. 7H).

**Fig. 8:**
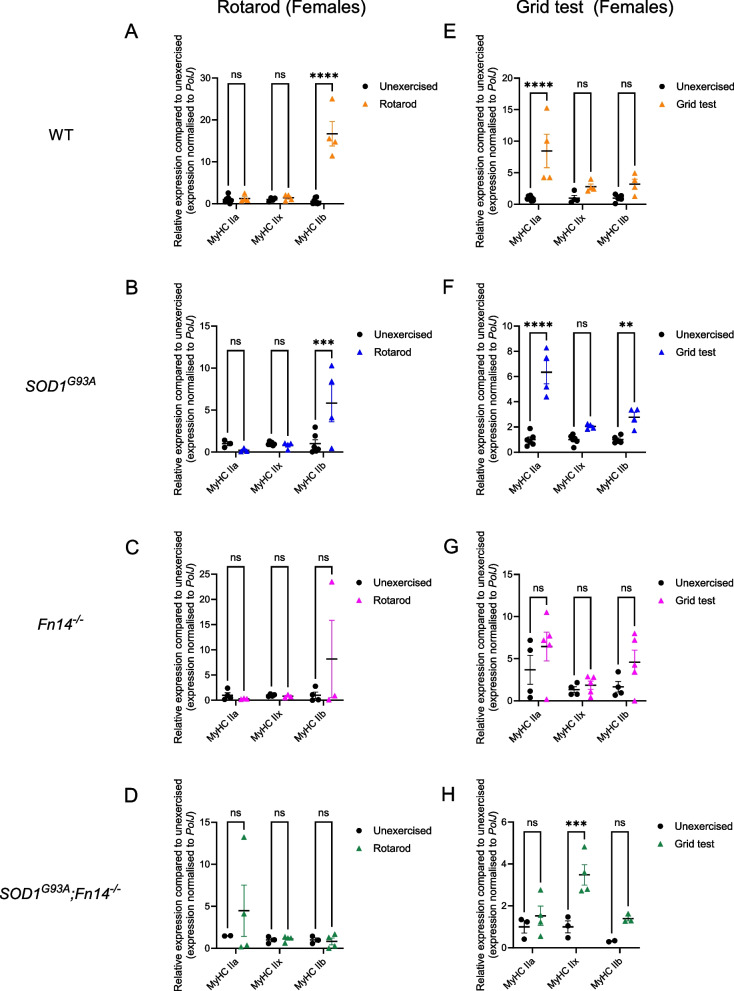
Type of exercise and genotype impact the expression of *MyHC* isoforms *IIa*, *IIx* and *IIb* in female mice. 12-week-old WT, *Fn14*^*−/−*^, *SOD1*^*G93A*^ and *SOD1*^*G93A*^*;Fn14*^*−/−*^ females were either placed on the rotarod (A-D) or performed the grid test (E–H) daily for 5 consecutive days. *Tibialis anterior* (TA) muscles were harvested approximately 2 h after the last bout of exercise. **A-D** qPCR analysis of *MyHC IIa*, *IIx* and *IIb* mRNA expression in unexercised and rotarod-exercised WT (**A**), *SOD1*^*G93A*^ (**B**), *Fn14*^*−/−*^ (**C**) and *SOD1*^*G93A*^*;Fn14*^*−/−*^ (**D**) females. Data are scatter dot plot mean ± SEM, *n* = 4–8 animals per experimental group, two-way ANOVA, ns = not significant, ***p < 0.001. ***p < 0.0001. **E–H** qPCR analysis of *MyHC IIa*, *IIx* and *IIb* mRNA expression in unexercised and grid test-exercised WT (**E**), *SOD1*^*G93A*^ (**F**), *Fn14*^*−/−*^ (**G**) and *SOD1*^*G93A*^*;Fn14*.^*−/−*^ (**H**) females. Data are scatter dot plot mean ± SEM, *n* = 3–8 animals per experimental group, two-way ANOVA, ns = not significant, ***p < 0.001. ***p < 0.0001

In rotarod-trained females, we observed a significant increased expression of only *MyHC IIb* in both rotarod-trained WT and *SOD1*^*G93A*^ mice compared to unexercised animals (Fig. 8A, B). In contrast, the expression of all three *MyHC* isoforms remained unchanged in rotarod-trained *Fn14*^*−/−*^ and *SOD1*^*G93A*^*;Fn14*^*−/−*^ animals compared to unexercised mice (Fig. 8C, D).

Finally, in grid test-trained females, only the expression of *MyHC IIa* was significantly increased in grid test-trained WT animals compared to unexercised mice (Fig. 8E) while the levels of both *MyHC IIa* and *IIb* were significantly elevated in grid test-trained *SOD1*^*G93A*^ mice compared to unexercised animals (Fig. 8F). The expression of all three *MyHC* isoforms remained unchanged in grid test-trained *Fn14*^*−/−*^ animals compared to unexercised mice (Fig. 8G). In *SOD1*^*G93A*^*;Fn14*^*−/−*^ animals, we observed a significant increase only in *MyHC IIx* expression compared to unexercised mice (Fig. 8H).

Combined, our results suggest that the rotarod and grid test exercise regimens did not lead to changes in MyHC isoform expression in all cases. Indeed, similar to our analyses of molecular effectors of the TWEAK/Fn14 signalling pathway and atrogenes, our data points to the influence of sex, genotype, exercise type and disease state on the expression of *MyHC IIa*, *IIx* and *IIb* isoforms in the TA muscle (summarised in Table 2).

**Table 2:**
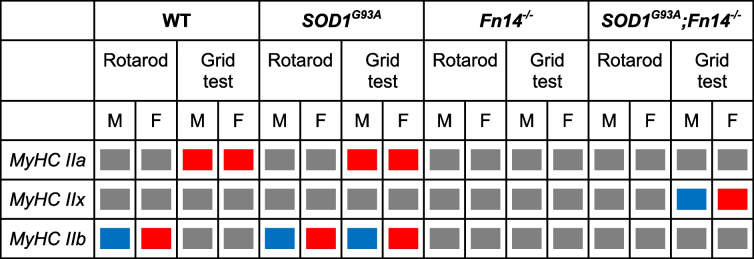
Effect of exercise, sex and genotype on expression of the myosin heavy chain isoforms

Red = upregulated

Blue = downregulated

Grey = no change

M = Males

F = Females

### Fn14 depletion and exercise influence myofiber size

We next determined if Fn14 depletion impacted myofiber size in the gastrocnemius muscle of rotarod- or grid test-trained 12-week-old WT, *Fn14*^*−/−*^, *SOD1*^*G93A*^ and *SOD1*^*G93A*^*;Fn14*^*−/−*^ males and females.

**Fig. 9:**
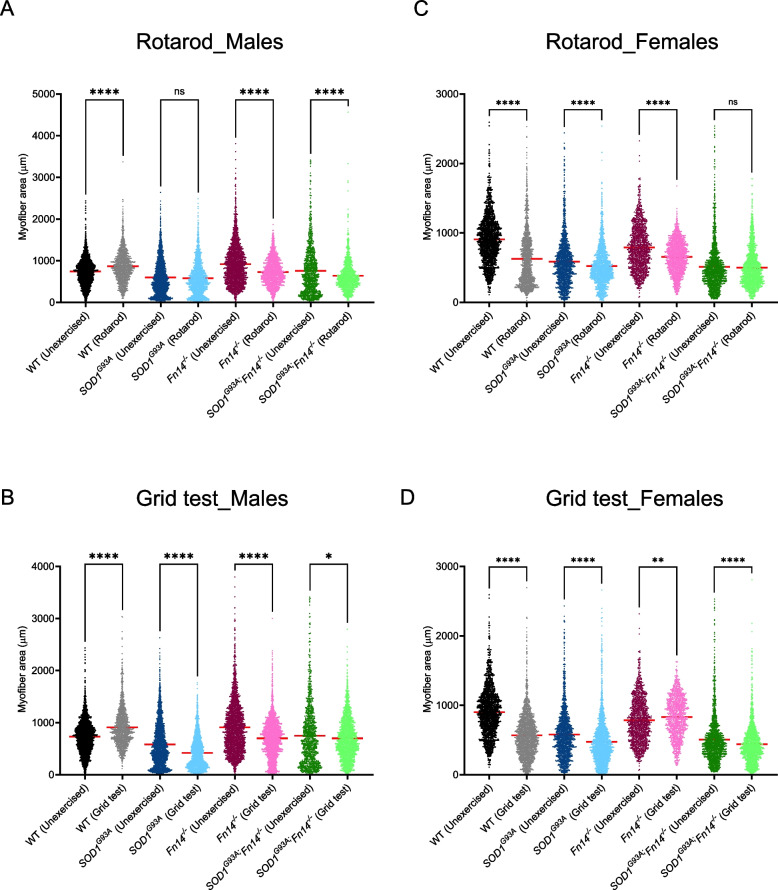
Type of exercise, genotype and sex impact muscle fibre size. 12-week-old WT, *Fn14*^*−/−*^, *SOD1*^*G93A*^ and *SOD1*^*G93A*^*;Fn14*^*−/−*^ males and females either performed the grid test or were placed on the rotarod daily for 5 consecutives days. Gastrocnemius muscles were harvested approximately 2 h after the last bout of exercise. **A** Quantification of myofiber area of laminin-stained cross-sections of gastrocnemius muscles from 12-week-old unexercised and rotarod-exercised WT, *Fn14*^*−/−*^, *SOD1*^*G93A*^ and *SOD1*^*G93A*^*;Fn14*^*−/−*^ males. Data are dot plot and mean, *n* = 3–4 animals per experimental group (> 1100 myofibers per experimental group), two-way ANOVA, ns = not significant, ****p < 0.0001. **B** Quantification of myofiber area of laminin-stained cross-sections of gastrocnemius muscles from 12-week-old unexercised and grid test-exercised WT, *Fn14*^*−/−*^, *SOD1*^*G93A*^ and *SOD1*^*G93A*^*;Fn14*^*−/−*^ males. Data are dot plot and mean, *n* = 3–4 animals per experimental group (> 1100 myofibers per experimental group), two-way ANOVA, *p < 0.05, ****p < 0.0001. **C** Quantification of myofiber area of laminin-stained cross-sections of gastrocnemius muscles from 12-week-old unexercised and rotarod-exercised WT, *Fn14*^*−/−*^, *SOD1*^*G93A*^ and *SOD1*^*G93A*^*;Fn14*^*−/−*^ females. Data are dot plot and mean, *n* = 3–4 animals per experimental group (> 1400 myofibers per experimental group), two-way ANOVA, ns = not significant, ****p < 0.0001. **D** Quantification of myofiber area of laminin-stained cross-sections of gastrocnemius muscles from 12-week-old unexercised and grid test-exercised WT, *Fn14*^*−/−*^, *SOD1*^*G93A*^ and *SOD1*^*G93A*^*;Fn14*^*−/−*^ females. Data are dot plot and mean, *n* = 3–4 animals per experimental group (> 1100 myofibers per experimental group), two-way ANOVA, **p < 0.01, ****p < 0.0001

Following rotarod training in males, we observed a significant increase in myofiber size of rotarod-trained WT animals compared to unexercised WT mice while this type of exercise did not impact myofiber size in *SOD1*^*G93A*^ animals (Fig. 9A). Interestingly, the myofiber size of rotarod-trained *Fn14*^*−/−*^ and *SOD1*^*G93A*^*;Fn14*^*−/−*^ mice was significantly smaller than in unexercised control animals (Fig. 9A), indicating that the combination of Fn14 depletion and rotarod exercise can reduce muscle size.

Similar to rotarod-trained WT males, grid test-trained WT males displayed a significant increase in myofiber size compared to unexercised animals (Fig. 9B). However, unlike rotarod-trained *SOD1*^*G93A*^ males, grid test-trained *SOD1*^*G93A*^ animals had a significant decrease in myofiber size compared to unexercised *SOD1*^*G93A*^ mice (Fig. 9B). A significant decrease in myofiber area was also observed in grid test-trained *Fn14*^*−/−*^ and *SOD1*^*G93A*^*;Fn14*^*−/−*^ mice compared to unexercised control animals (Fig. 9B), suggesting that both the *SOD1*^*G93A*^ genotype and Fn14 depletion negatively impact muscle size following a resistance exercise regimen.

Interestingly, rotarod training in females resulted in a significant decrease in myofiber size in all rotarod-trained experimental groups when compared to unexercised animals (Fig. 9C).

In grid test-females, a significant decrease in myofiber size was also observed in grid test-trained WT, *SOD1*^*G93A*^ and *SOD1*^*G93A*^*;Fn14*^*−/−*^ mice compared to unexercised animals (Fig. 9D). However, a significant increase in myofiber size was found in grid test-trained *Fn14*^*−/−*^ animals compared to unexercised mice (Fig. 9D).

Together, our data points to independent influences of exercise type, sex and genetics on muscle fiber size.

## Discussion

In this study, we aimed to better understand how increased Fn14 expression in an ALS mouse model with chronic denervation and muscle wasting could contribute to muscle pathology and disease progression. To achieve this, we genetically deleted *Fn14* in both WT and *SOD1*^*G93A*^ mice and observed behavioural, molecular and histological changes that were dependent on exercise, sex and disease progression.

In the first instance, we not only confirmed our previous observation of increased expression of Fn14 in the skeletal muscle of *SOD1*^*G93A*^ ALS mice during disease progression [33], but we also validated the previously reported negative correlation between the activity of the TWEAK/Fn14 pathway and the expression of the known molecular and metabolic effectors *Glut4*, *Klf15*, *HKII* and *PGC-1*α [29]*.* Interestingly, we recently demonstrated a similar but inverse negative correlation in the skeletal muscle of another neuromuscular mouse model, SMA mice, whereby the expression levels of *Tweak* and *Fn14* decreased during disease progression while those of *Glut4*, *Klf15*, *HKII* and *PGC-1*α increased [32]. One important distinction between these two studies is the developmental stage investigated. Indeed, SMA mice were of pre-weaned age [32] while the *SOD1*^*G93A*^ ALS mice were at adult stages (current study), suggesting that the TWEAK/Fn14 signalling pathway is differentially regulated at different stages of muscle development. This differential regulation might have an impact on downstream metabolic requirements and regulation as well as therapeutic interventions in cases of dysregulation.

One of our key findings is the extended lifespan of Fn14-depleted *SOD1*^*G93A*^ ALS mice, which is contrary to the absence of impact following genetic *Tweak* deletion in the same mouse model, which we have previously reported [33]. This suggest that the detrimental effect of the aberrant activity of the TWEAK/Fn14 pathway in skeletal muscle of *SOD1*^*G93A*^ ALS mice is driven by the receptor (Fn14) and not the ligand (TWEAK). This aligns with previous work that points to a greater role for Fn14 than TWEAK in enabling pathway activity [34]. It is also possible that the differential impacts observed in TWEAK- and Fn14-depleted *SOD1*^*G93A*^ ALS mice are due to Fn14-independent TWEAK signalling [56] and/or TWEAK-independent Fn14 signalling [57]. Furthermore, the distinct effects of TWEAK and Fn14 depletion in *SOD1*^*G93A*^ ALS mice could further be caused by their known roles in other tissues such as the heart, gastrointestinal tract, kidney, liver, central nervous system and epithelium [58–60]. As the genetic knock-out of TWEAK and Fn14 was systemic in both cases, we cannot exclude additional benefits or detrimental effects stemming from altered function in other cells and tissues. Regardless of the reasons, our combined studies point to a greater therapeutic value in modulating Fn14 over TWEAK.

In addition to lifespan, we also observed improvements in skeletal muscle pathology at molecular and histological levels in Fn14-depleted *SOD1*^*G93A*^ mice. These changes did not occur in *Fn14*^*−/−*^ mice when compared to WT animals, suggesting that the effects were dependent on disease stage. Interestingly, we have previously shown increased muscle fibre and NMJ endplate sizes in TWEAK-depleted *SOD1*^*G93A*^ ALS mice [33], further supporting a role for the TWEAK/Fn14 pathway in muscle pathology in this mouse model and in more general adult denervation-induced muscle atrophy [34]. Of note is that in both TWEAK- and Fn14-depleted *SOD1*^*G93A*^ ALS mice, there were no significant improvements in motor function [33], suggesting that simply targeting the TWEAK/Fn14 pathway is not sufficient for the recovery of the neuromuscular unit.

Surprisingly, the beneficial impact of Fn14 depletion on the survival of *SOD1*^*G93A*^ ALS mice was almost masked when the mice underwent weekly rotarod and grid test assessments for approximately 16 weeks as the enhanced physical activity itself had a positive impact on survival of the *SOD1*^*G93A*^ ALS mice. Interestingly, there are reports of both beneficial and detrimental effects of exercise in ALS mouse models and patients that suggest that exercise regimen (type and length) and sex are important factors that contribute to the observed outcomes [61–65]. In our study, short weekly bouts of grid test and rotarod were sufficient to improve survival. Further investigations showed that the combination of 5 consecutive days of exercise (rotarod or grid test) and Fn14 depletion was sufficient to induce changes at molecular and histological levels in the skeletal muscle of 12-week-old animals. These changes were dependent on disease stage, exercise and sex. Combining exercise and Fn14 depletion may therefore lead to potentially, additive, synergistic and/or antagonistic interactions that may be dependent on the exercise regimen itself and individual characteristics. However, in our study, we did not identify clear commonalities between Fn14 depletion and exercises that would point to shared mechanisms. Furthermore, the results of some analyses such as myofiber size and expression of atrogenes did not necessarily align with the increased survival in Fn14-depleted and exercised *SOD1*^*G93A*^ mice, suggesting more complex and possibly multi-systemic mechanisms that influence overall disease progression in these mice.

One key observation was that changes in *Tweak* and *Fn14* expression appeared dependent on the type of exercise, sex and genotype of the animal. For example, *Fn14* levels displayed a differential expression in *SOD1*^*G93A*^ males only, whereby it was increased following rotarod and decreased following the grid test. Conversely, in females, *Fn14* levels specifically increased in WT mice following both rotarod and grid test, when compared to unexercised animals. These diverse patterns may reflect the complex metabolic adaptations impacted by disease, Fn14 presence/absence, sex and type of exercise. Typically, endurance exercises promote the use of aerobic/oxidative metabolic pathways in skeletal muscle while resistance exercises favour anaerobic/glycolytic metabolic pathways [66]. In ALS, skeletal muscle metabolism during rest and exercise is altered in both pre-clinical models and patients [67–70], which could alter how ALS muscle adapts to different types of exercises and the overall beneficial vs detrimental outcomes [62, 63]. As for Fn14, it is typically increased in skeletal muscle of healthy individuals and adult mice following exercise, irrespective of type (endurance vs resistance) [71–74]. Conversely, the muscle-specific deletion of Fn14 and the ubiquitous TWEAK deletion in mice both improved exercise capacity and oxidative metabolism [75, 76], suggesting that sustained and/or aberrant increase in TWEAK/Fn14 activity expression during exercise may be detrimental. It is therefore unclear why the expression of both the ligand and effector are commonly reported as being elevated following exercise. Of note, we only observed changes in *Fn14* expression in exercised WT female mice in our study, which may be due to our selected exercise regimens (length and type of exercise). Nevertheless, our results, combined with previous studies, suggest and support a complex interaction between Fn14 regulation, disease state, exercise and the metabolic status of muscle.

Another noticeable result is the influence of genotype, sex and exercise on the expression of the atrogenes *MuRF-1* and *Atrogin-1*. For example, we found that the expression of *MuRF-1* is significantly elevated in *SOD1*^*G93A*^ males following both the rotarod and grid test, supporting previous studies on the negative impact of exercise in ALS patients [63, 77]. In *SOD1*^*G93A*^*;Fn14*^*−/−*^ males, *MuRF-1* levels remained low in both rotarod and grid test groups, aligning with the previous report of reduced neurogenic muscle atrophy in muscle-specific Fn14-depleted animals [75]. However, in females, *MuRF-1* expression was significantly increased in WT mice only following the rotarod and all experimental groups after the grid test. While the differential expression patterns of both atrogenes might appear contradictory, previous studies have demonstrated that their regulation can be controlled by distinct pathways and in a sex-dependent fashion [78–82]. Of note, our analysis of muscle fibres shows an absence of perfect correlation between the expression of atrogenes and myofiber size, suggesting that changes in *MuRF-1* and *Atrogin-1* are not sufficient to improve muscle size and that other molecular effectors and regulatory pathways may be responsible for modulating muscle mass [83]. Indeed, Fn14 has previously been demonstrated to modulate myoblast fusion [84, 85], a process that contributes to muscle size growth during regeneration, which typically occurs following bouts of exercise. As such, Fn14 depletion may have affected myofiber size in a subset of our rotarod- and grid test-trained animals.

The expression of molecular and metabolic effectors previously shown to be regulated by TWEAK/Fn14 signalling also appear to be dependent on genotype, sex and type of exercise. For example, *PGC-1α* expression was upregulated in both the rotarod- and grid test-trained *SOD1*^*G93A*^ males and Fn14 depletion restored the levels to normal only in the grid-test exercised *SOD1*^*G93A*^*;Fn14*^*−/−*^ mice. In females however, *PGC-1α* levels were significantly elevated in rotarod- and grid-test trained WT animals and Fn14 depletion restored the levels to normal only in the rotarod-trained WT females. These differential patterns and relationships between genotype, exercise, sex and metabolic effectors most likely result from the combination of different metabolic pathways favoured by different types of exercise [66], as well as the impact of ALS-causing mutations and sex on muscle metabolism [67–70, 86, 87]. Indeed, the previously reported role of the TWEAK/Fn14 pathway in the regulation of PGC-1α and mitochondrial content in skeletal muscle [88] may have contributed to our PGC-1a observations with added influences from sex, genotype and exercise that still need to be explored.

MyHC isoforms IIa, IIx and IIb are specific to fast twitch skeletal muscles such as the TA and they each confer distinct metabolic and functional properties to skeletal muscle fibres [89]. Furthermore, skeletal muscle can adapt rapidly to metabolic changes induced by exercise, which can be reflected by altered MyHC expression [90]. In our study, we indeed observed some changes in MyHC isoform expression that were dependent on exercise type, genotype and sex, suggesting that the different combinations of these factors had distinct effects on the properties of skeletal muscle. In some instances (e.g. grid test-trained *SOD1*^*G93A*^*;Fn14*^*−/−*^ males), changes in the same direction of more than one MyHC isoform was observed, a phenomenon previously reported following a short bout of exercise [91].

Finally, our analyses of myofiber size further emphasized the differential responses of skeletal muscle from females and males to types of exercise, disease state and Fn14 depletion. In fact, throughout our molecular and histological analyses, sex-dependent differences were observed. These align with previous studies showing the effect of sex on responses to exercise [80, 81, 87], in *Fn14*^*−/−*^ mice exposed to neonatal hypoxia–ischemia [92], on general muscle properties [93], on expression of MyHC isoforms [94] and on disease onset and response to exercise in *SOD1*^*G93A*^ mice [95]. All of these extrinsic and intrinsic factors are therefore important to consider when assessing skeletal muscle adaptations in ALS. While our work provides some interesting insights, it is important to note its key limitations. Firstly, the impact of exercise on *SOD1*^*G93A*^*;Fn14*^*−/−*^ mice was observed in animals that performed both types of exercise weekly from 8 weeks of age to humane endpoint. However, the rotarod and grid test experiments were done separately on 12-week-old animals for 1 week only. Furthermore, our study focused on the known metabolic effectors downstream of TWEAK and Fn14, which means that additional genes and signalling cascades could be impacted by exercise, sex and/or genotype and contribute to our observed results. Finally, our research was aimed at investigating skeletal muscle but as Fn14 depletion is systemic, some of the beneficial and detrimental effects reported may be due to other cells and tissues.

## Conclusions

Our study provides additional insights on the role of the TWEAK/Fn14 pathway in a denervation-induced muscle pathology as modelled in the *SOD1*^*G93A*^ ALS mice. Importantly, we demonstrate that the benefits of Fn14 depletion are impacted by exercise and sex. This is particularly relevant in the context of the current therapeutic landscape of the ALS field, where combinatorial therapies that include exercise regimens are being explored by many research and clinical teams. As such, a better understanding and consideration of the interactions between treatments, muscle metabolism, exercise and sex will be of importance in future studies.

## Supplementary Information

Additional file 1: Supplementary Figure 1. Survival of *SOD1*^*G93A*^*;Fn14*^*+/->*^ and are not significantly different. Survival curves of *SOD1*^*G93A*^*;Fn14*^*+/-*^ and *SOD1*^*G93A*^*; Fn14*^*-/-*^ mice that performed both the rotarod and grid test weekly from 8 weeks to humane endpoint (males and females combined). Data are represented as Kaplan-Meier survival curves, *n*= 12-13 animals per experimental group, Log-rank (Mantel-Cox), ns = not significant.

Additional file 2: Supplementary Table 1. Mouse primers used for quantitative real-time PCR.

## Data Availability

All data generated or analysed during this study are either included in this published article [and its supplementary information files] or are available from the corresponding author on reasonable request.

## Abbreviations

α-BTX: α-Bungarotoxin
ALS: Amyotrophic lateral sclerosis
C9ORF72: Chromosome 9 open reading frame 72
ChAT: Choline acetyltransferase
FBS: Fetal bovine serum
Fn14: Fibroblast growth factor inducible 14
FUS: Fused in Sarcoma
Glut4: Glucose transporter 4
HKII: Hexokinase II
Klf15: Krüppel-like factor 15
MyHC: Myosin heavy chain
NeuN: Neuronal nuclear antigen
NF- κB: Nuclear factor kappa-light-chain-enhancer of activated B cells
PBS: Phosphate buffered saline
PFA: Paraformaldehyde
PGC-1α: Peroxisome proliferator-activated receptor-gamma coactivator 1α
PolJ: RNA polymerase II polypeptide J
SDS: Sodium dodecyl-sulfate
SMA: Spinal muscular atrophy
SOD1: Superoxide dismutase 1
TA: Tibialis anterior
TDP-43: TAR DNA-binding protein 43
TWEAK: Tumor necrosis factor-like weak inducer of apoptosis
