# Supplementary figures and images for: Exercise, disease state and sex influence the beneficial effects of Fn14-depletion on survival and muscle pathology in the SOD1G93A amyotrophic lateral sclerosis (ALS) mouse model
Source: Skelet Muscle. 2024 Oct 14;14:23. doi: 10.1186/s13395-024-00356-0 (PMC11472643; doi:10.1186/s13395-024-00356-0)

ns [ 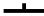  $SOD1^{G93A};Fn14^{+/-}$   
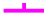  $SOD1^{G93A};Fn14^{-/-}$

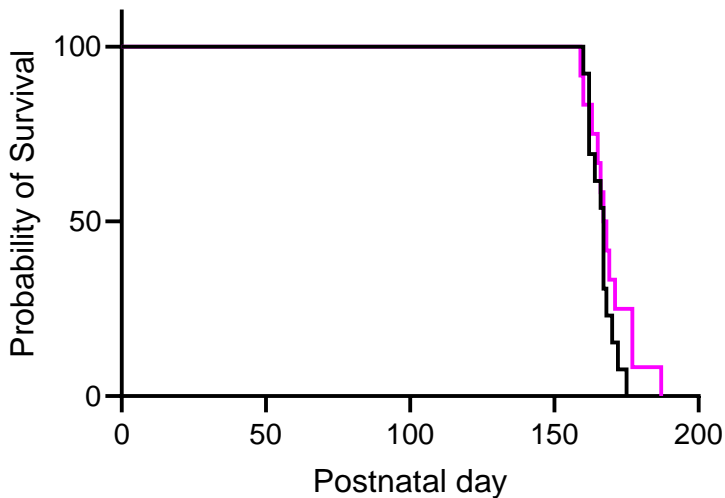

Supplement: Supplementary file 1 — Additional file 1: Supplementary Figure 1. Survival of SOD1G93A;Fn14+/-> and are not significantly different. Survival curves of SOD1G93A;Fn14+/- and SOD1G93A; Fn14-/- mice that performed both the rotarod and grid test weekly from 8 weeks to humane endpoint (males and females combined). Data are represented as Kaplan-Meier survival curves, n= 12-13 animals per experimental group, Log-rank (Mantel-Cox), ns = not significant. [file 13395_2024_356_MOESM1_ESM.pdf]
