# Supplementary material for: Exercise, disease state and sex influence the beneficial effects of Fn14-depletion on survival and muscle pathology in the SOD1G93A amyotrophic lateral sclerosis (ALS) mouse model
Source: Skelet Muscle. 2024 Oct 14;14:23. doi: 10.1186/s13395-024-00356-0 (PMC11472643; doi:10.1186/s13395-024-00356-0)
Supplement: Supplementary file 2 — Additional file 2: Supplementary Table 1. Mouse primers used for quantitative real-time PCR. [file 13395_2024_356_MOESM2_ESM.docx]

**Supplementary Table 1. Mouse primers used for quantitative real-time PCR.**

| Gene | Forward | Reverse |
| --- | --- | --- |
| *Atrogin-1* | 5’-TCAAAGGCCTCACGATCACC-3’ | 5’-CCTCAATGACGTATCCCCCG-3’ |
| *Fn14* | 5′-TCGTGTTGGGATTCGGCTTGGT-3' | 5′-ACTTTTCTCTCCGGCGGCATCT-3' |
| *Glut4* | 5′-GACGGACACTCCATCTGTTG-3' | 5′-CATAGCTCATGGCTGGAACC-3' |
| *HKII* | 5′-GAAGGGGCTAGGAGCTACCA-3' | 5′-CTCGGAGCACACGGAAGTT-3' |
| *Klf15* | 5′-TGCGTCGGCACACAGGCGAGAA-3' | 5′-CCGGTGCCTTGACAACTCATCT-3' |
| *MuRF-1* | 5′-AGGACTCCTGCCGAGTGAC-3' | 5′-TTGTGGCTCAGTTCCTCCTT-3' |
| *PGC-1α* | 5′-TGGAGTGACATAGAGTGTGCTGC-3' | 5′-CTCAAATATGTTCGCAGGCTCA-3' |
| *PolJ* | 5′-ACCACACTCTGGGGAACATC-3' | 5′-CTCGCTGATGAGGTCTGTGA-3' |
| *Tweak* | 5′-AAGTTCACTGAGGGGCCTTGCT-3' | 5′-TGTGAACAAGCTCTGGCTGCCT-3' |
| *Osteopontin* | 5’-GACAACAACGGAAAGGGCAG-3’ | 5’-GATCGGCACTCTCCTGGCT-3’ |
| *ChAT* | 5’-GGTGGCCCAGAAGAGCAGTATC-3’ | 5-’ATTGGAGGCAGGCGTTCATC-3’ |
| *NeuN* | 5’-GAGGAGTGGCCCGTTCTG-3’ | 5’-AGGCGGAGGAGGGTACTG-3’ |
| *MyHC IIa* | 5’-AAGCGAAGAGTAAGGCTGTC-3’ | 5’-GTGATTGCTTGCAAAGGAAC-3’ |
| *MyHC IIx* | 5’-CCAAGTGCAGGAAAGTGACC-3’ | 5’-AGGAAGAGACTGACGAGCTC-3’ |
| *MyHC IIb* | 5’-ACAAGCTGCGGGTGAAGAGC-3’ | 5’-CAGGACAGTGACAAAGAACG-3’ |
